# Synthesis and Characterization of New-Type Soluble β-Substituted Zinc Phthalocyanine Derivative of Clofoctol

**DOI:** 10.3390/molecules28104102

**Published:** 2023-05-15

**Authors:** Sabrine Dridi, Jamel Eddine Khiari, Gabriele Magna, Manuela Stefanelli, Larisa Lvova, Federica Mandoj, Khaoula Khezami, Mahmut Durmuş, Corrado Di Natale, Roberto Paolesse

**Affiliations:** 1 Experimental Sciences and Supramolecular Chemistry, Laboratory of Didactic Research, Higher Institute of Education and Continuing Training (ISEFC), University of Tunis El Manar, Tunis 1002, Tunisia; sabrinegandour@gmail.com; 2 Experimental Sciences and Supramolecular Chemistry, Laboratory of Didactic Research, Higher Institute of Education and Continuing Training (ISEFC), University of Carthage, Tunis 1054, Tunisia; jamelkhiari@yahoo.fr; 3 Department of Chemical Science and Technologies, University of Rome Tor Vergata, 00133 Rome, Italy; gabriele.magna@uniroma2.it (G.M.); manuela.stefanelli@uniroma2.it (M.S.); larisa.lvova@uniroma2.it (L.L.); federica.mandoj@uniroma2.it (F.M.); 4 Department of Chemistry, Faculty of Engineering and Natural Sciences, Istinye University, 34396 Istanbul, Turkey; khaoula@gtu.edu.tr; 5 Department of Chemistry, Gebze Technical University, 41400 Kocaeli, Turkey; durmus@gtu.edu.tr; 6 Department of Electronic Engineering, University of Rome Tor Vergata, 00133 Rome, Italy; dinatale@uniroma2.it

**Keywords:** phthalocyanines, solubility, gas sensors, quartz microbalances

## Abstract

In this work, we have described the synthesis and characterization of novel zinc (II) phthalocyanine bearing four 2-(2,4-dichloro-benzyl)-4-(1,1,3,3-tetramethyl-butyl)-phenoxy substituents on the peripheral positions. The compound was characterized by elemental analysis and different spectroscopic techniques, such as FT-IR, ^1^H NMR, MALDI-TOF, and UV-Vis. The Zn (II) phthalocyanine shows excellent solubility in organic solvents such as dichloromethane (DCM), n-hexane, chloroform, tetrahydrofuran (THF), and toluene. Photochemical and electrochemical characterizations of the complex were performed by UV-Vis, fluorescence spectroscopy, and cyclic voltammetry. Its good solubility allows a direct deposition of this compound as film, which has been tested as a solid-state sensing material in gravimetric chemical sensors for gas detection, and the obtained results indicate its potential for qualitative discrimination and quantitative assessment of various volatile organic compounds, among them methanol, n-hexane, triethylamine (TEA), toluene and DCM, in a wide concentration range.

## 1. Introduction

Phthalocyanines (Pcs) are synthetic tetrapyrrolic macrocycles that have been extensively studied due to their unique properties, such as their aromatic 18 π-electronic structure, planarity, high symmetry, good thermal stability, and efficient light absorption [1,2]. Pcs and their metal complexes (MPcs) show green and blue colors, leading to their first use as dyes and pigments [3]. They have also been exploited in sensors, electronic displays, non-linear optical devices, solar cells, semiconductors, data storage systems, catalysts, and photodynamic therapy (PDT) [4]. One relevant requirement for Pcs applications is their solubility, which can allow their purification and consequent exploitation in target devices. However, unsubstituted phthalocyanines have very low solubility in common organic solvents [5], so a decisive ambition of Pc chemistry is to obtain soluble derivatives. This aim can be achieved by introducing appropriate functional groups at the peripheral positions of the phthalocyanine ring and selecting the metal ion coordinated at the inner core [6]. Therefore, the modification of π-conjugated system size by alternating the type, number, and positions of the substituents on the phthalocyanine frameworks enhances their solubility and affect their aggregation in solutions [7], which is generally related to intermolecular interactions such as π-–π stacking, hydrogen–bond, acid–base interactions, and donor–acceptor interactions [8,9]. In this respect, it is possible to design Pcs that have high solubility by introducing bulky groups either on peripheral or non-peripheral positions [10]. Avoiding aggregation leads to soluble phthalocyanines, making them good candidates for particular applications.

**Scheme 1:**
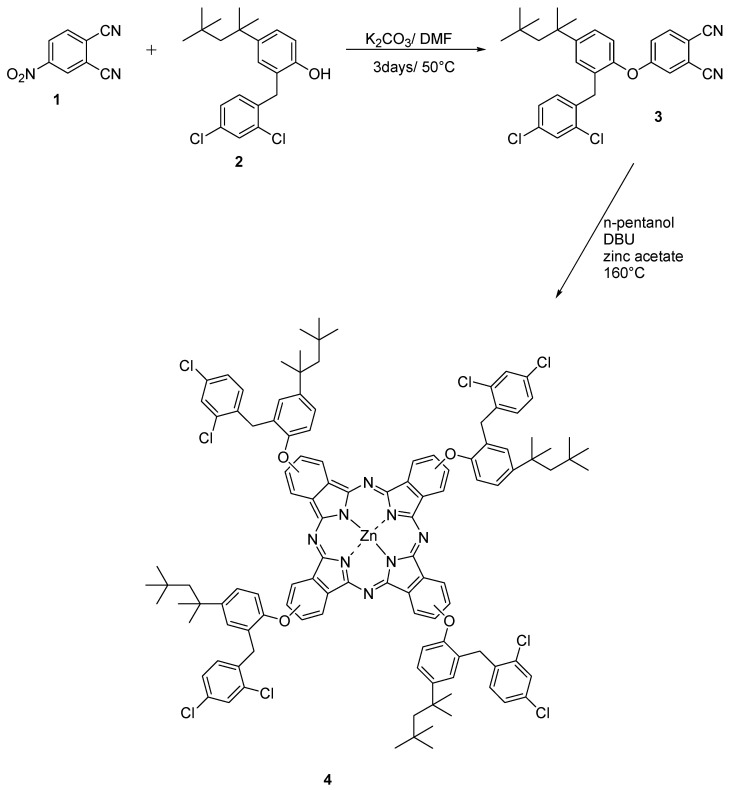
Synthetic route of complex **4**.

In this paper, we report the synthesis of zinc phthalocyanine containing 2-(2,4-dichloro-benzyl)-4-(1,1,3,3-tetramethyl-butyl)-phenoxy moiety starting from the Clofoctol derivative, which is a synthetic compound used in the treatment of bacterial respiratory infections and exhibits antiviral activity against SARS-CoV-2 (see **3** and **4** in Scheme 1) [11]. Solubility, aggregation, fluorescence, electrochemical, and optical properties of the complex were determined, and gravimetric sensors investigated its possible use as a sensing material. In particular, two sensors were produced by depositing both titled and Ni phthalocyanines onto Quartz Microbalances (QMBs), and a comparison between their chemical sensitivities toward volatile organic compound (VOC) vapors was made.

## 2. Results

### 2.1. Synthesis and Characterization

The procedure for the synthesis of **3** and **4** is given in Scheme 1. The preparation of phthalonitrile **3** was performed by nucleophilic aromatic nitro displacement of 2-(2,4-dichloro-benzyl)-4-(1,1,3,3-tetramethyl-butyl) phenol (**2**) with 4-nitrophthalonitrile in the presence of K_2_CO_3_ in dry DMF [12]. Following the usual protocol for the synthesis of tetrasubstituted Pcs, **4** was achieved in dry pentanol by cyclotetramerization reaction of phthalonitrile **3** in the presence of anhydrous Zn(OAc)_2_ and 1,8-diazabicyclo[5.4.0]undec-7-ene (DBU) base [13]. The symmetric Pc derivative **4** was easily purified by column chromatography, with a satisfying yield of 58%.

### 2.2. Spectroscopic Characterization

The structures of compounds **3** and **4** were checked by employing elemental analysis and common spectroscopic techniques such as FT-IR, ^1^H NMR, ^13^C NMR, MALDI-TOF, and UV-Vis. According to FT-IR spectral data, **3** showed its vibration band at 2229 cm^−1^, indicating the presence of the -C≡N group (Figure S1). The ^1^H NMR spectrum of **3** proves the disappearance of the OH signal of the starting phenol, with the appearance of further aromatic protons confirming the occurrence of the substitution (Figure S1). The structure of compound **3** was identified within the reflectron mode by matrix-assisted laser desorption/ionization time-of-flight mass (MALDI-TOF) spectrometry using 2,5-dihydroxybenzoic acid (DHB) as matrix. The presence of potassium ion K^+^ was detected in the molecular ion peak at 530.42 as [M + K]^+^ (Figure S2). All spectral data collected support the proposed structure of **3**.

The formation of **4** was confirmed in the FT-IR spectra by the disappearance of the sharp C≡N vibration of precursor **3** at 2238 cm^−1^ (Figure S1). The ^1^H NMR spectrum of **4** suffers from signal broadening, probably due to aggregation at the concentration for NMR measurements, although the pattern is similar to that of compound **3** [14]. The MALDI-TOF spectrum indicated that the molecular weight value of this phthalocyanine is in accordance with the suggested molecule. The molecular ion peak of compound **4** was observed at 2032 as [M+H]^+^ (Figure S3).

### 2.3. UV-Vis Characterization

**Figure 1:**
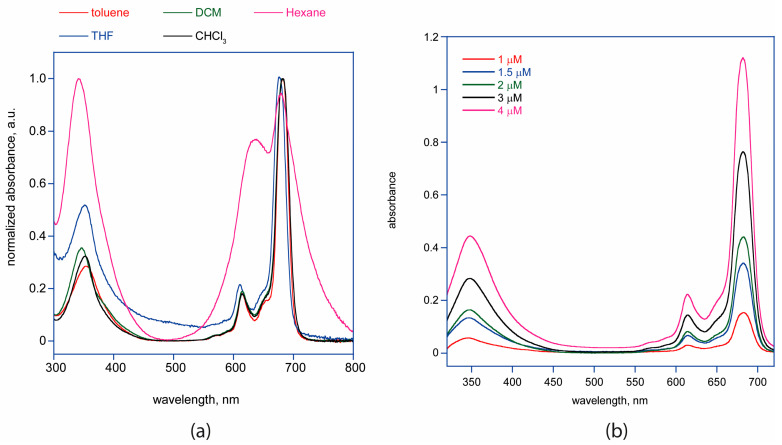
(**a**) UV–Vis spectra of **4** in different solvents. (**b**) **4** in DCM at different concentrations.

UV-Vis absorption spectroscopy is the most effective technique for proving the formation of Pcs. Pcs generally display two strong absorption bands in their ground-state absorption spectra. The first is the B band observed between 300–500 nm in the UV region, and the other is the Q band placed at 600–750 nm in the visible region, showing a more intense absorption [15]. Figure 1a shows the UV-Vis absorption spectra of **4** in different solvents. In the UV-Vis absorption spectra of **4**, an intense Q band absorption was observed at 682 nm, while the B band absorption was observed around 354 nm. Metallophthalocyanines that have D4h symmetry exhibit only an intense Q band absorption in their UV-Vis spectra [16]. The synthesized phthalocyanine **4** showed good solubility in most of the common organic solvents, such as toluene, THF, DCM, chloroform, and hexane, although in this latter solvent, the UV-Vis spectrum of **4** shows a broadening of bands with a relative lowering of the main Q-band, suggesting the presence of macrocycle aggregation.

UV-Vis absorption spectra in DCM (see spectra in Figure 1b) at different concentrations show that the main Q-band of **4** has a high molar extinction coefficient of 3.14 × 10^5^ M^−1^cm^−1^ at 683 nm in DCM, with good linearity observed for the Lambert–Beer law, confirming the absence of aggregation (R^2^ = 99.79).

**Figure 2:**
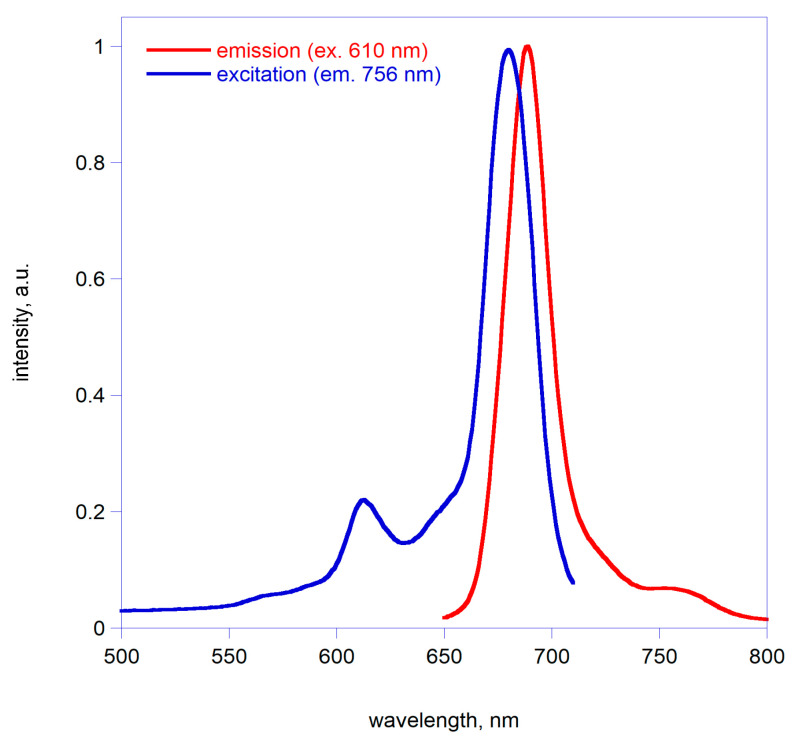
Excitation and emission spectra of **4** in DCM.

As a further confirmation of the solubility of the macrocycle, fluorescence measurements have been recorded in DCM. Figure 2 shows the fluorescence emission and excitation spectra for **4**. In the case of emission spectra, excitation was set at 610 nm, corresponding to the main Q-band peak, and emission was recorded in the 650–800 nm range. On the other hand, in the case of excitation spectra, emission was recorded at 756 nm scanning excitation wavelength in the 500–710 nm interval. Complex **4** is fluorescent, with the excitation spectrum superimposable to the absorption spectrum for **4** in DCM. Furthermore, the fluorophore showed no photodegradation during the experiments due to light excitation.

### 2.4. Electrochemical Studies

**Figure 3:**
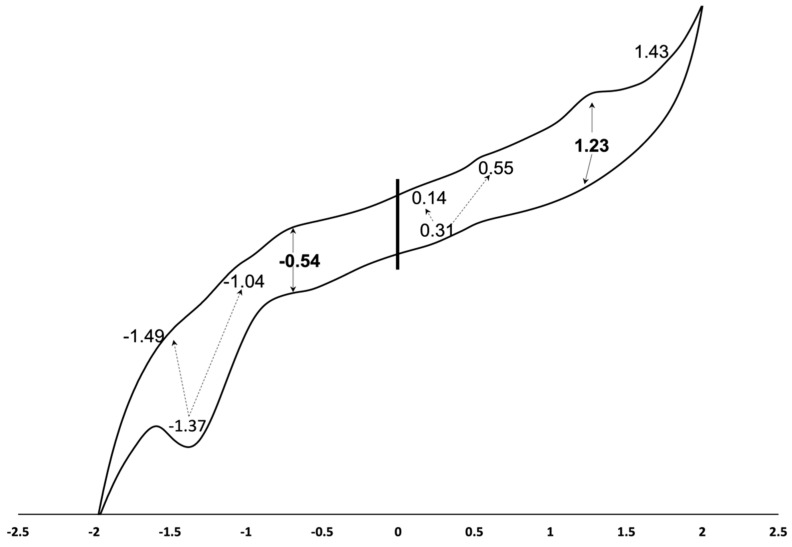
Cyclic voltammograms of 5 × 10^−5^ M of complex **4** in DCM and 0.1 M TBAClO_4_ solution.

The investigation of the electrochemical behavior of the newly synthesized complex **4** is important to predict the possible usage in different technological areas. To characterize the degree of electronic interactions among the Zn(II)Pc and 2-(2,4-dichloro-benzyl)-4-(1,1,3,3-tetramethyl-butyl)-phenoxy peripheral substituents in complex **4**, we investigated the electrochemical properties of this macrocycle by cyclic voltammetry (CV) technique. The CV of **4** was recorded in the potential range from −2.0 to 2.0 V, and the details on complex **4** redox behavior are summarized in Figure 3.

In the cathodic region, Zn(II)Pc **4** undergoes one well-defined one-electron process at −0.54 V and one split two-electron reduction at −1.37 V followed by two small backway oxidation waves at −1.49 V and at −1.04 V, respectively. In the anodic region, the oxidation waves at 0.43 V and 1.23 V are well seen and correspond to the data on Zn(II)Pc electrochemical behavior reported in the literature [17,18,19]. Thus, for instance, for octa-3-hydroxypropylthio substituted Zn(II) phthalocyanine, the first oxidation wave was registered at 0.49 V, and the second one was observed at 0.98 V, and the processes have been assigned to the metal- and ring-based processes [1]. An additional irreversible small oxidation peak at 1.43 V and the split oxidations at 0.11 V and 0.55 V reflect the complex **4** redox behavior registered in the cathodic region and may be assigned to the influence of the peripheral substituents on the electron transfer to the phthalocyanine center.

The splitting of redox processes indicates that the peripheral substituents of complex **4** behave as equivalent but not interacting reaction centers. The HOMO-LUMO gap of compound **4** evaluated as the potential difference between the first oxidation and first reduction corresponds to the high value of 2.6 V, which is indicative of the high inter-molecular electron transfer abilities and potential of the developed compound as a sensing semiconductor material for both electron-donating and electron-withdrawing analytes [20,21]. Zn(II)Pc **4** gives a ring-based redox process only due to the electro-inactive nature of the Zn(II) metal coordinated to the macrocycle, as the energy level of the d orbital of the zinc is placed out of the HOMO-LUMO energy levels [22]. This result may also be due to the smaller atomic radius of Zn(II) metal compared to other metal atomic radii such as Co and Mn [17].

### 2.5. Gas Sensing Properties

The detection of VOCs vapors plays an important role in various fields, such as agriculture, medicine, and the food industry, since they are widely used and may provoke respiratory difficulties and irritation effects [23]. As gas-sensing materials, phthalocyanines have revealed high sensitivity to large sets of organic vapors [24,25], even if solubility and limited sensitivity often hinder their implementation in broader applications. The evaluation of the sensing properties of phthalocyanine for the detection of VOCs has received a lot of interest. Therefore, many types of research have been conducted on the sensing performance of Pc-based devices and the effect of environmental conditions on their performances [26,27,28]. Unlike the usual methods based on conductometric devices, in the present study, we deposited the sensing material onto a gravimetric sensor, such as Quartz Microbalances (QMBs). The sensing performances of **4** were then explored by depositing 15 kHz of material onto each of the two electrodes of a QMB. Subsequently, the sensor has been exposed to different VOCs, utilizing a series of mass flow controllers.

**Figure 4:**
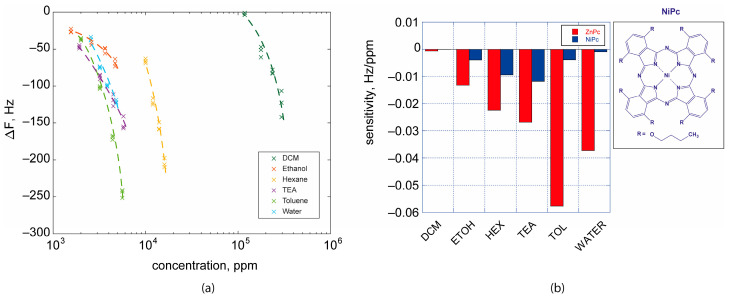
(**a**) Characteristic curves of QMB sensor based on **4**. Data are reported on a semilogarithmic scale due to the wide range of VOC concentrations. (**b**) Sensitivities calculated as the first derivative of linear fitting equations of data in the range of concentration measured. In the case of NiPc data, and experimental details were previously reported in [25].

Several concentrations have been obtained from liquid samples in bubblers diluting the saturated vapors of the analytes with pure nitrogen in order to obtain the desired concentrations. By using this approach, it is possible to dilute vapors in the headspace of bubblers up to 1:100. Saturated vapors at 20 °C were calculated accordingly to Antoine equations (parameters can be found at https://webbook.nist.gov/chemistry/, accessed on 1 March 2023). As probe analytes, we selected dichloromethane, ethanol, n-hexane, triethylamine (TEA), toluene, and water due to the different interactions that may occur with receptors in the film. Usually, the Linear Solvation Equation Relationship (LSER) reports five parameters that account for electron donor, electron acceptor, dipole–dipole, dipole–”induced dipole”, and “induced dipole”–”induced dipole” interactions. The sensor exhibited high variations in oscillation frequency toward most of the volatile compounds tested, especially toluene and water (see Figure 4a). This first outcome suggests that the macrocycle preferentially interacts with guest molecules by π–π interactions (toluene) or π–hydrogen bonds (water), likely due to the additional aromatic units conferred by the four clofoctol appended units. At the same time, this functional unit is supposed to favor dispersion interactions, such as in the case of hexane. Finally, the presence of Zn(II) metal facilitates the coordination with molecules bearing electron-donating groups, as in the case of amines. Considering the sensitivity as the first derivative of the response curves, it is possible to extrapolate the selective pattern of the sensor. Remarkably, if we compare the sensitivity of **4** Pc with other phthalocyanines previously studied in similar experiments by our group, such as a NiPc derivative [25] (1,4,8,11,15,18,22,25-octabutoxy-Ni(II)-phthalocyanine), we can observe how the sensitivity of the novel synthesized material is from four to ten times higher (see Figure 4b). This outcome suggests that this new phthalocyanine derivative can be extremely useful in the sensor field firstly because it made easy the deposition of films onto QMBs or transducer surface directly from a chloroform solution thanks to the good solubility of this macrocycle. Furthermore, this class of phthalocyanine derivatives showed to be a receptor with improved sensitivity toward a large class of compounds, and a potentially excellent porphyrinoid-based material for expanding the cross-sensor selectivity in electronic nose platforms, for example.

## 3. Materials and Methods

### 3.1. Equipment

All reagents were purchased from Merck or Aldrich and used without further purification. 4-Nitrophthalonitrile was synthesized and purified according to the protocol mentioned in the literature [29]. Volatile Organic Compounds (VOC) were reagent grade from Sigma Aldrich and used as received. Fluorescence experiments were carried out by SHIMADZU RF-1501 spectrofluorimeter with automatized internal working settings. UV-Vis spectra were performed by an Agilent Cary 60 spectrophotometer.

Voltammetric measurements were performed by PalmSens3 potentiostat/galvanostat (PALMSENS BV, Utrecht, The Netherlands) on disk Pt WE (2.5 mm diameter) versus SCE reference electrode (Amel, Milan, Italy) and Pt wire counter electrode (Sigma-Aldrich, Darmstadt, Germany) in 5.0 × 10^−5^ M solution of 4 in dichloromethane containing 0.1 M tetrabutylammonium perchlorate salt as background electrolyte. The CVs were registered in the −2.0 to +2.0 V range with a scan rate of 100 mV/s. The FT-IR spectra were recorded on a Perkin Elmer 1600 FT-IR spectrophotometer using KBr pellets. ^1^H-NMR spectra were recorded on a Bruker 700 MHz spectrometer in CDCl_3_ and chemical shifts were reported (δ) relative to Me_4_Si as internal standard. Mass spectra of the compounds were measured on a Micromass Quattro LC/ULTIMA LC-MS/MS spectrometer and MALDI-MS in dihydroxybenzoic acid as MALDI matrix using nitrogen laser accumulating 50 laser shots using Bruker Microflex LT MALDI-TOF mass spectrometer Bremen, Germany. The elemental analyses were performed on a Costech ECS 4010 instrument. FT-IR, MALDI-MS, ^1^H NMR, and ^13^C-NMR spectra of compounds **3** and **4** are reported in the Supporting Information (Figures S1–S5).

Analytes were delivered in the vapor phase by a gas flow system, including MKS mass flow meter and Channel Readout mass controllers.

### 3.2. Sensors Preparation and Characterization

Quartz microbalance (QMBs) were AT-cut quartzes oscillating at 20 MHz (KVG GmbH). In QMBs sensors, the resonance frequency is inversely proportional to the mass adsorbed onto the electrode surface. QMB utilized has a nominal and experimental mass sensitivity of about 7 Hz/ng [30]. The drop casting technique was used for sensing layer deposition, ensuring a total frequency shift of approximately 30 kHz. An electronic oscillator circuit acquired the fundamental oscillation frequency of the quartz each second. The sensor was tested with vapors of different volatile compounds, pouring liquid samples in bubblers at a constant temperature of 303 K. The VOC concentration can be estimated using the Antoine law; parameters of the volatile compound are available in the NIST database (available at www.nist.gov/webbook, accessed on 1 March 2023). The measurement protocol has an initial baseline phase under nitrogen for acquiring the baseline frequencies of the sensor, an exposure phase during which a known concentration of analyte vapor is fluxed in the measurement chamber containing the sensor, and a final desorption phase where nitrogen flow allows the desorption of analyte molecules from the sensing films. The frequency variations between the end of the measurement phase and the baseline value were chosen as sensor response features. Each volatile compound was measured at four different concentrations, and each concentration was randomly measured three times.

### 3.3. Synthesis

#### 3.3.1. 4-[2-(2,4-Dichloro-benzyl)-4-(1,1,3,3-tetramethyl-butyl)-phenoxy]-phthalonitrile (**3**)

Clofoctol **2** (0.4 g, 1.15 mmol) was dissolved in 10 mL dry DMF. An excess of anhydrous K_2_CO_3_ was added to the mixture. Then 4-nitrophthalonitrile **1** (0.2 g, 1.15 mmol) was added to the mixture. The reaction mixture was continually stirred powerfully under the N_2_ atmosphere at 50 °C for 3 days. After the reaction mixture was cooled down at room temperature, it was poured into 100 mL of ice-water media. A creamy white precipitate was formed; it was filtered and then washed with water until the filtrate became neutral. The white product was recrystallized from methanol, filtered, and finally dried under vacuum forming a white purified powder. Yield 0.32 g (57%), mp 211–213 °C; FT-IR ν_max_/cm^−1^ (KBr): 3042 (Ar–H), 2229 (C≡N), 1565, 1469, 1251 (Ar–O–C), 1097, 956, 834, 823; ^1^H NMR (700 MHz, CDCl_3_, δ ppm): 7.67 (d, *J* = 8.7 Hz, 1H), 7.39–7.35 (m, 1H), 7.30 (d, *J* = 9.4 Hz, 2H), 7.15–7.08 (m, 2H), 7.07 (s, 1H), 6.96 (d, *J* = 8.2 Hz, 1H), 6.92 (d, *J* = 8.4 Hz, 1H), 3.97 (s, 2H), 1.75 (s, 2H), 1.40 (s, 6H), 0.76 (s, 9H). ^13^C-NMR (CDCl_3_, 125 MHz, δ ppm): 31.11, 32.68, 33.50, 38.56, 56.87, 108.62, 114.20, 117.23, 120.17, 127.79, 129.38, 130.93, 131.30, 132.49, 134.68, 135.68, 135.74, 148.82, 161.52. Elemental analysis C_29_H_28_Cl_2_N_2_O: 70.87 C%, 5.74 H%, 5.70 N%; found: 70.96 C%, 5.73 H%, 5.54 N%. MALDI-TOF (+) (*m*/*z*): Calc. for C_29_H_28_Cl_2_N_2_O 491.46; found 530.42 [M + K]^+^.

#### 3.3.2. 2(3),9(10),16(17),23(24)-Tetrakis 2-(2,4-dichloro-benzyl)-4-(1,1,3,3-tetramethyl-butyl)-phenoxy zinc (II) phthalocyanine (**4**)

Compound **3** (0.1 g, 0.2 mmol) and few drops of 1,8-diazabicyclo [5.4.0] undec-7-ene (DBU) in dry pentanol (3.00 mL) with anhydrous Zn(OAc)_2_ (0.018 g, 0.1 mmol) were added in round bottom flask and heated with efficient stirring at 160 °C under inert N_2_ atmosphere for 16 h. The green–blue color was examined during the progress of time. The observed green–blue product was cooled down to room temperature when the mixture was diluted with methanol. The obtained crude product was washed several times with methanol, water to eliminate the excessive impurities. Finally, the product was purified by silica gel chromatography (Ethyl acetate-hexane) and crystallized from methanol to obtain the target macrocycle. Yield 60 mg (58%); mp > 300 °C; C_116_H_112_Cl_8_N_8_O_4_Zn; FT-IR ν_max_/cm^−1^ (KBr): 3020, 1553, 1450, 1250, 1284, 1104, 925, 830, 760; ^1^H NMR (700 MHz, CDCl_3_, δ ppm): 7.58–6.97 (m, 36H), 4.30–4.05 (m, 8H), 1.84–1.70 (m, 8H), 1.50–1.34 (m, 24H), 0.93–0.67 (m, 36H). Elemental analysis C_116_H_112_Cl_8_N_8_O_4_Zn: 68.59 C%, 5.56 H%, 5.52 N%; found: 68.56 C%, 5.53 H%, 5.54 N%. MALDI-TOF (+) (*m*/*z*): Calc. C_116_H_112_Cl_8_N_8_O_4_Zn 2031.20; found 2032.82 [M+H]^+^.

## 4. Conclusions

The main goal of this study was to investigate the possible use of novel soluble zinc(II) phthalocyanine **4** as a sensing material. The major advantage of this compound is its solubility in organic solvents and the absence of aggregation in the concentration range studied. Zinc(II) phthalocyanine **4** shows fluorescence properties that should be used, in turn, to investigate the sensing properties of this fluorophore in solution. This property indicates that the compound can potentially be used in medical applications. The electrochemical properties of the proposed compound were also investigated because of its possible potential usage in electro-catalysis, electro-sensing, and electrochromic devices.

## Data Availability

Data available upon request.

## Supplementary Materials

The following supporting information can be downloaded at: https://www.mdpi.com/article/10.3390/molecules28104102/s1, Figure S1: FT-IR spectrum of compounds **3** and **4**; Figure S2: MALDI-TOF mass spectrum of compound **3**; Figure S3: MALDI-TOF mass spectrum of compound **4**; Figure S4: ^1^H NMR spectrum of **3** in CDCl_3_, at 298 K; Figure S5: ^13^C NMR spectrum of **3** in CDCl_3_, at 298 K.

## Sample Availability

Sample of compound 4 available from the authors upon request.
