# Supplementary material for: Synthesis and Characterization of New-Type Soluble β-Substituted Zinc Phthalocyanine Derivative of Clofoctol
Source: Molecules. 2023 May 15;28(10):4102. doi: 10.3390/molecules28104102 (PMC10223450; doi:10.3390/molecules28104102)
Supplement: Supplementary file 1 [file molecules-28-04102-s001.zip › molecules-2398119-supplementary.pdf]

# Supporting Information

# Synthesis and Characterization of New-Type Soluble β-Substituted Zinc Phthalocyanine Derivative of Clofoctol

**Sabrine Dridi [1], Jamel Eddine Khiari [2], Gabriele Magna [3], Manuela Stefanelli [3], Larisa Lvova [3], Federica Mandoj [3], Khaoula Khezami [4,5], Mahmut Durmuş [5], Corrado Di Natale [6] and Roberto Paolesse [3,*]**

[1] Experimental Sciences and Supramolecular Chemistry, Laboratory of Didactic Research, Higher Institute of Education and Continuing Training (ISEFC), University of Tunis El Manar, Tunis 1002, Tunisia; sabrinegandour@gmail.com

[2] Experimental Sciences and Supramolecular Chemistry, Laboratory of Didactic Research, Higher Institute of Education and Continuing Training (ISEFC), University of Carthage, Tunis 1054, Tunisia; jamelkhiari@yahoo.fr

[3] Department of Chemical Science and Technologies, University of Rome Tor Vergata, 00133 Rome, Italy; gabriele.magna@uniroma2.it (G.M.); manuela.stefanelli@uniroma2.it (M.S.); larisa.lvova@uniroma2.it (L.L.); federica.mandoj@uniroma2.it (F.M.)

[4] Department of Chemistry, Faculty of Engineering and Natural Sciences, Istinye University, 34396 Istanbul, Turkey; khaoula@gtu.edu.tr

[5] Department of Chemistry, Gebze Technical University, 41400 Kocaeli, Turkey; durmus@gtu.edu.tr

[6] Department of Electronic Engineering, University of Rome Tor Vergata, 00133 Rome, Italy; dinatale@uniroma2.it

* Correspondence: roberto.paolesse@uniroma2.it

# Table of Contents

| | Page |
|---|---|
| **Figure S1.** FT-IR spectrum of compounds **3** and **4** | 2 |
| **Figure S2.** MALDI-TOF mass spectrum of compound **3** | 2 |
| **Figure S3.** MALDI-TOF mass spectrum of compound **4** | 3 |
| **Figure S4.** $^{1}$H NMR spectrum of **3** in $CDCl_3$, at 298 K. | 3 |
| **Figure S5.** $^{13}$C NMR spectrum of **3** in $CDCl_3$, at 298 K. | 4 |

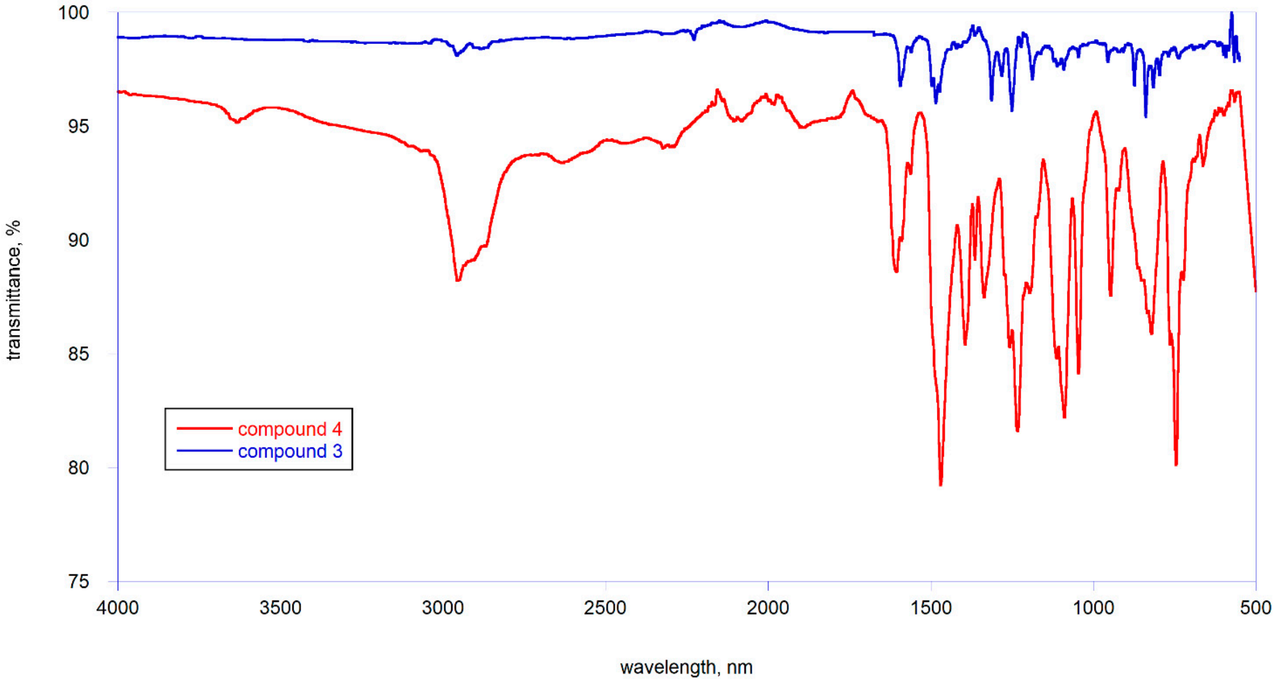


Figure S1. FT-IR spectrum of compounds **3** (blue line) and **4** (red line)

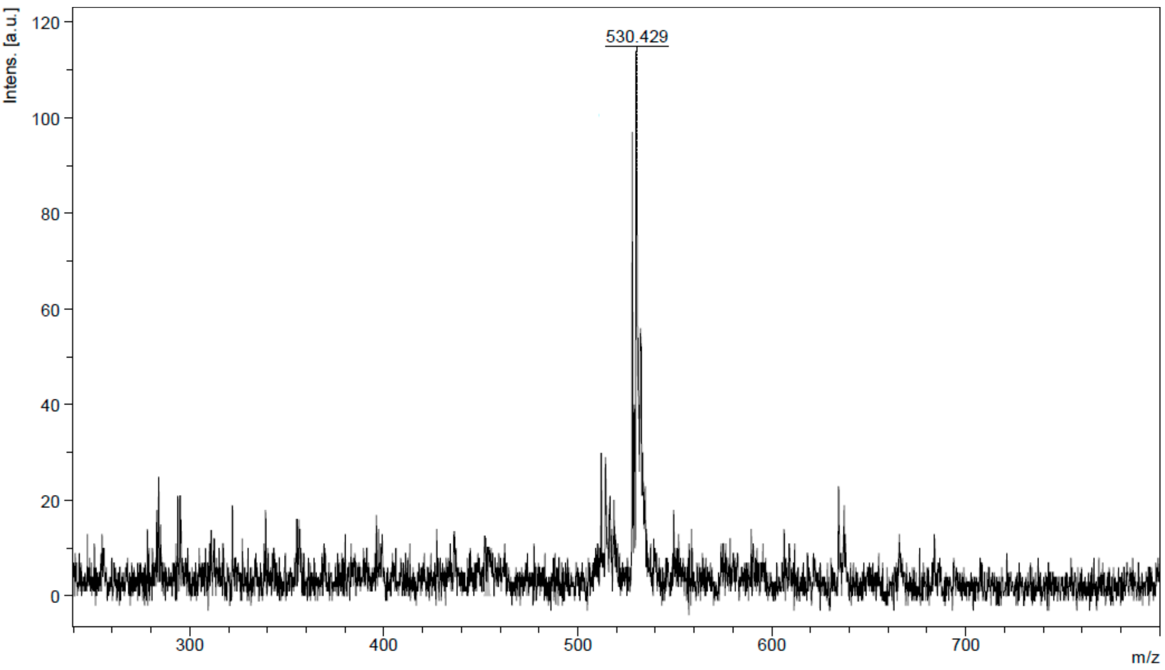


Figure S2. MALDI-TOF mass spectrum of compound **3**

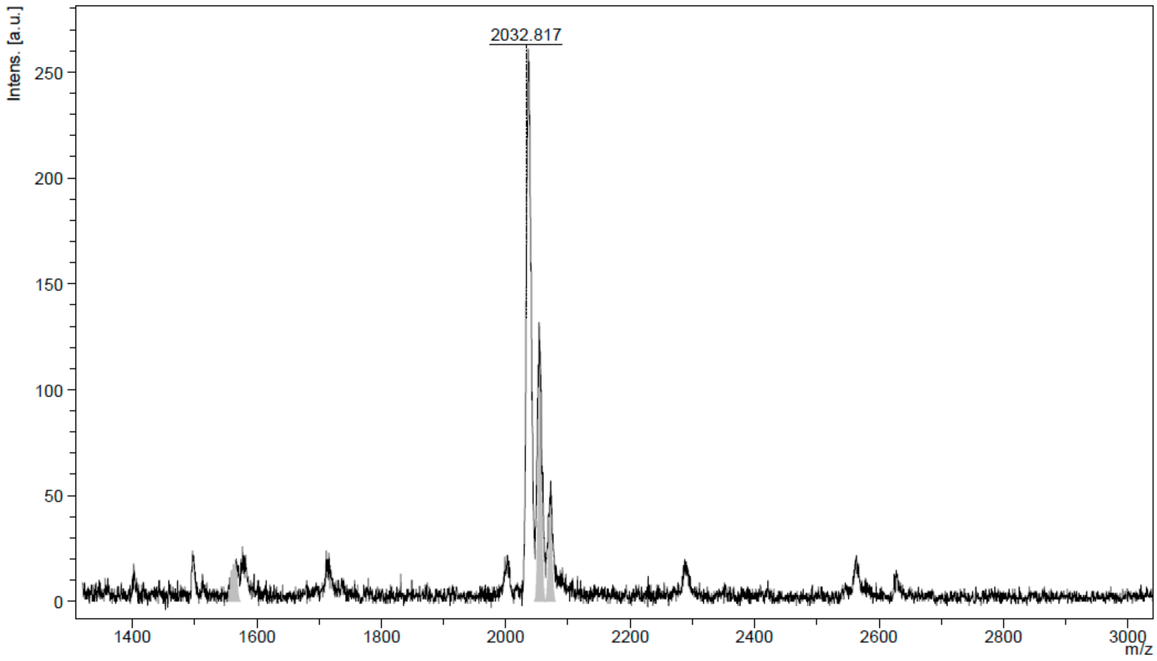


Figure S3. MALDI-TOF mass spectrum of compound **4**

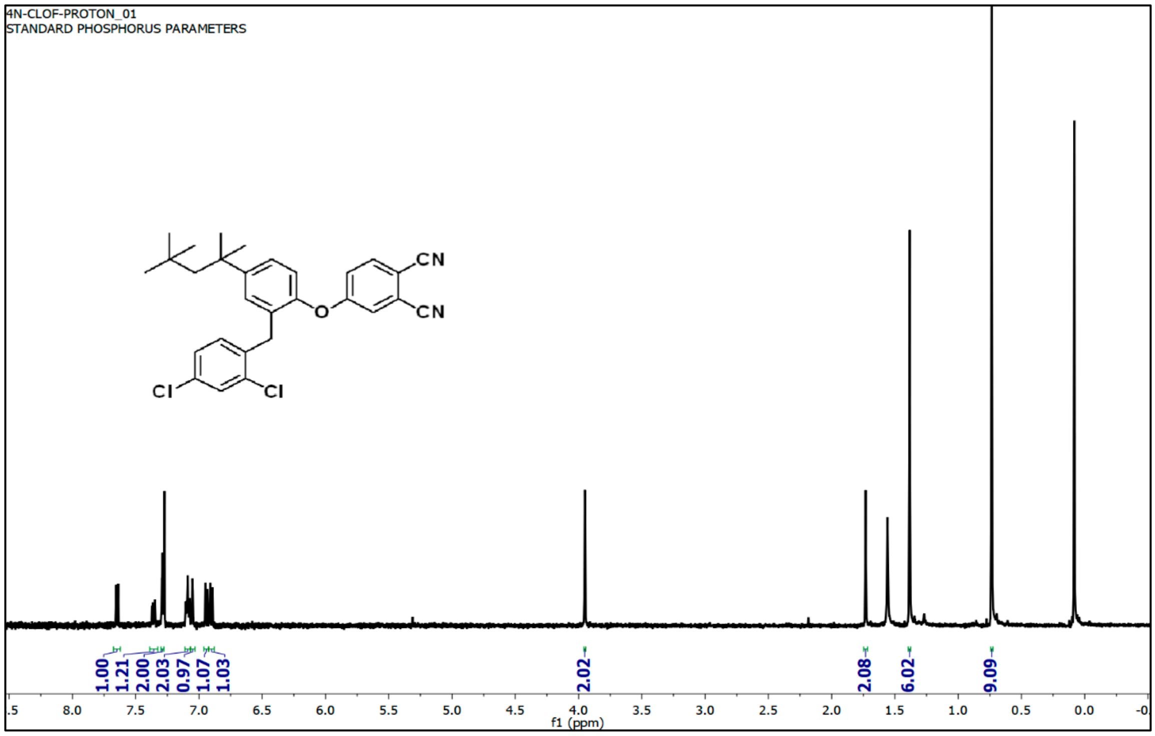


Figure S4. $^{1}$H-NMR spectrum of compound **3** in $CDCl_3$

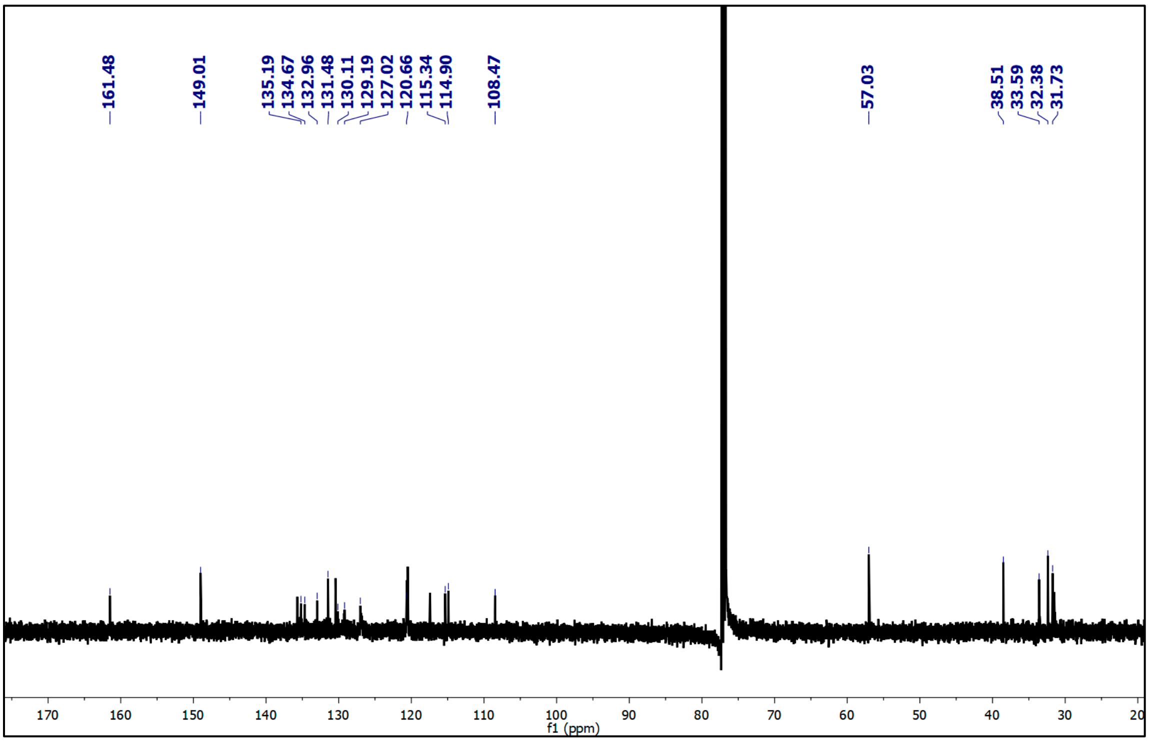


Figure S5. ${}^{13}$C-NMR spectrum of compound **3** in $CDCl_3$
